# Comparison of Laboratories Directors’ and Assessors’ Opinions on Challenges and Solutions of Standardization in Iran: A Qualitative Study

**DOI:** 10.5539/gjhs.v7n4p358

**Published:** 2015-01-25

**Authors:** Hamid Ravaghi, Nazanin Abolhassani

**Affiliations:** 1 School of Health Management and Information Sciences, Iran University of Medical Sciences, Tehran, Iran; 2 Department of Health Management and Economics, School of Public Health, Tehran University of Medical Sciences, Tehran, Iran

**Keywords:** standardization, medical laboratory, assessors, laboratory directors

## Abstract

**Objective:**

The quality medical laboratory services play a vital role in healthcare systems. Iran has set national standards based on the international standard ISO15189. These standards came into force in September 2007. Given the important role of both laboratories professional and assessors in the standardization, this study aims to compare and analyze medical laboratory directors’ and assessors’ opinions about this process, its challenges and relevant solutions.

**Methods:**

This qualitative study was conducted on two populations in 2013. The first survey population consisted of 150 assessors. The second group consisted of directors working in medical laboratory settings. From all universities of medical sciences, 258 medical laboratories were randomly selected. Data were gathered using two open-ended questionnaires and analyzed using the thematic analysis.

**Results:**

Challenges and relevant solutions regarding the standardization and standards, the assessment process and assessor, laboratories, external entities and contextual factors across laboratories directors and assessors were derived and compared. Both groups had a positive attitude towards the standardization process. However, they expressed some concerns regarding the process and accordingly proposed solutions to overcome the challenges.

**Conclusion:**

This study provides insights into the challenges and solutions of the standardization from two professional groups’ viewpoint. These two factors are closely related and should be considered when implementing standards since a positive perception of them increases the likelihood of successful standardization. Similarities and divergences regarding challenges and solutions of the standardization, in turn, can provide insights into how this process can be improved and deserve policy makers’ attention to continue the progress.

## 1. Introduction

Medical laboratory services play a vital role in healthcare systems; (Spitzenberger & Edelhäuser, 2006) high-quality and reliable laboratory services are necessary for effective and well-functioning health systems (Cobbina, Agbezudor, Amuzu, & Gyampomah, 2012). Nowadays, many national and international quality standards exist for laboratory practices. These standards are lists of requirements that need to be met in order to ensure quality practice (Datema, Oskam, & Klatser, 2011). Many countries have adopted internationally accepted standards such as ISO17025 and/or ISO15189 (Nkengasong, 2010). Other countries, such as Iran, have developed their own standards based on the country-specific circumstances. The ISO standards used by the Reference Health Laboratory (RHL) as source documents in drafting national standards came into force in September 2007 and all medical laboratories were required to apply these standards (Dahim et al., 2009; Anjarani, 2013). In Iran, there are 52 medical science universities which supervise the delivery of all health services in their catchment areas. The General Directorate of Laboratory Affairs (GDLA) in each medical sciences university is responsible for laboratory assessments and licensing using their employed assessors (Dahim et al., 2009).

The standardization process involves participation from frontline staff to senior management and their perceptions on this process are hence important. On the other hand, the core of the accreditation process is assessors recruited to conduct quality assessments using relevant standards. Their role in facilitating continuous quality improvement is also increasingly recognized (Greenfield & Braithwaite, 2008; Plebani, 2001). So, understanding and comparing the perspectives of both groups are essential to the successful implementation of the standards.

To this end, the perspectives of both groups were examined to uncover similarities and divergences in the standardization within and across medical laboratory directors and assessors. This research is the first to compare and analyze the opinions of medical laboratory directors and assessors about the standardization process, its challenges and relevant solutions to use standards in Iran. This in turn can provide insights into how this process can be improved.

## 2. Methods

This qualitative study was conducted on two populations in 2013. The first survey population consisted of all 150 assessors employed by the GDLA in each university of medical sciences who were selected from 330 assessors using the RHL database. The second group consisted of directors working in medical laboratory settings. Using the RHL database, 258 medical laboratories from all universities of medical sciences were randomly selected. The laboratories were from public/private sectors and hospital-based/outpatient laboratories.

Data were gathered using two open-ended questionnaires derived from the review of literature and the experts’ opinion. To check the reliability and validity of the questionnaires, they were pilot tested by four medical laboratory directors and four assessors and minor changes were made accordingly. The questions were about the participants’ perceptions of challenges and solutions regarding the national standardization scheme, the assessment process and assessors, laboratories, external entities and contextual factors. The questionnaires were sent via mail along with an official invitation letter signed by the RHL director. The information sheet and consent form were provided. The ethics approval was obtained by Tehran University of Medical Sciences research ethics committee. The qualitative data were analyzed using the thematic analysis. The member check strategy was used and comments were incorporated in the final analysis. It helped to ensure that the findings were congruent with the participants’ perceptions and opinions.

## 3. Results and Discussion

Of the 150 questionnaires sent to assessors, 51 completed questionnaires were returned. The assessors working in GDLA completed the questionnaire. They had an average laboratory job experience of 15 years (range 15-22) and had worked as assessors for 4 years (range 2-6). In regard with educational certifications, most respondents (80%) were medical laboratory scientists. The medical laboratory directors had a doctoral degree in medical laboratory science, followed by clinical pathology (30%) and other laboratory specialties. The mean years of job experience was 20.8. Out of 258 questionnaires, 100 completed ones were received. There were 62 private outpatient laboratories, 20 public and 18 private hospital based laboratories in the study.

Data collected from the questionnaires were coded and accordingly and categorized into five themes regarding challenges and proposed solutions from the point of view of both assessors and laboratories directors were developed. The themes included attitudes towards the standardization and standards, the assessment process and assessor, laboratories, external entities and contextual factors. Similarities and divergences regarding challenges and solutions of the standardization across medical laboratory directors and assessors were then explored. These findings have been provided in Table 1.

**Table 1:** Themes extracted from the interviews

Themes	assessors		Laboratories directors	

	challenges	solutions	challenges	solutions
**Attitudes towards the standardization and standards**	Overly bureaucratic, time consuming, Inefficient, Costly process Increased workloads and stress for laboratory staffs	Harmonization among laboratories Revising the standards and related guidelines’ Adopting a staged approach for standardization Different operational plans according to laboratories’ resources	Costly process, Time-consuming High paper workload Lack of shared understanding about the standardization concept	Adopting a stepwise approach and a slower and more gradual way of implementation Updating and revising the standards, checklists and guidelines based
**The assessment process and assessor**	Differences between the assessment of hospital and outpatient laboratories/public and private laboratories Lack of motivation in public sector/Inadequate number of assessor/insufficient training Dissatisfaction/Difficulties in the interpretation of the standards Different levels of quality among laboratories and lack of harmonized assessment process	Clearly defined assessor selection criteria/The evaluation of assessment process and assessors Training/more number of assessors/Increasing assessors’ motivation and commitment/More interactions between the assessors and other stakeholders/Providing financial rewards and professional recognition for assessors	Lack of a shared understanding of the assessment concepts and purposes/Inadequate time needed for assessment Long gap between two consecutive assessments Lack of providing feedback or delayed feedback to the laboratories/Low number of experienced assessors/Inability to communicate, over strict and unfair assessment	Building an assessment culture Following a well-defined plan for assessments/Devoting more time to the assessment process Conducting assessments in several phases/Providing timely feedback to the laboratories/Creating a network among the assessors Recruitments of competent assessors/Fixed members of assessment teams for a specific period of time
**Laboratories**	Lack of motivation Few laboratories’ involvement in the standardization Inadequate related information Lack of support from laboratory senior management Weak quality culture Financial and human resources problems	Training of laboratory staff Building a culture of quality in laboratories The harmonization among laboratories	Low familiarity with the concepts of the standardization and quality improvement among laboratories Inertia and reluctance to change Pressure on the private laboratories High workload Inadequate number of laboratory personnel High rate of personnel turn over	More commitment and involvement of laboratory managers/Development of a motivation system/Training the staff or managers/Better communication with other laboratories/Establishing an internal audit system in laboratories/Participating in the external quality control program
**External entities**	Limited authorities of the GDLA’ Low involvement of professional association in the standardization Low executive authority of the GDLA	Involvement of professional associations in composition and revision of standards and guidelines, setting criteria for assessor selection evaluation of assessment process and training Providing educational courses by universities Adjoining the principles of quality management system to academic curriculum of laboratory sciences	Discrimination against private laboratories by the entities responsible for the accreditation Lack of an executive authority Unwillingness and unclear role of professional associations	Incentive mechanisms by the RHL to encourage the accredited laboratories/Establishment of a coalition among the professional associations/More participation of the professional associations Revising standards and checklists/Delegation of the assessment process to professional associations Providing technical assistance to laboratories/More involvement of the professional associations in the standardization
**Contextual factors**	Low laboratory tariff levels Inadequate policy-level support/Financial problems, high inflation rate and increased price of laboratory equipment/Lack of timely reimbursement to laboratories by the insurance organizations	Policy-level support for laboratory accreditation system Reasonable increase of laboratory tariff levels	Financial and economic issues High price of proper kits and equipment, maintenance services and the space and facilities needed to comply with the requirements of the standards	Setting reasonable laboratory tariffs Providing financial aid for the quality improvement program Allocating more budgets to medical laboratories

### 3.1 Attitudes Towards the Standardization and Standards

Both assessors and laboratories’ directors had a positive attitude towards the standardization process and acknowledged it as a tool for quality improvement. However, they expressed some concerns regarding the standardization process and accordingly proposed a range of solutions to overcome the challenges. Items considered by assessors as major challenges to the standardization and standards included ‘overly bureaucratic’, ‘time consuming’, ‘inefficient’, ‘costly’, and ‘increased workloads and stress for laboratory staffs’.

Although the laboratories’ directors had similar concerns regarding problematic items, they ranked these items differently. These findings are similar to those of a study, conducted by Gough and Reynolds in the UK, on clinical pathology accreditation (Gough & Reynolds, 2000). The laboratories’ directors rated ‘costly process’ as the most challenging obstacle, followed by ‘time consuming’, ‘high paper workload’, and ‘lack of shared understanding about the standardization concept’. The issue of cost as a barrier to standardization among laboratories is in consistence with findings of other studies (McGrowder, Crawford, Irving, Brown, & Anderson-Jackson, 2010; Zeh et al., 2010; Ravaghi et al.,2014). Moreover, the laboratories’ directors explicitly pointed to the challenge of the lack of shared understanding about the standardization concept which indicates that there were some difficulties in conveying the core concepts of standardization to laboratories’ professionals. A study in Japan also suggested that the standard’s concepts should be more clarified (Aoyagi & Kawai, 2006).

In the meantime, several key factors that seemed to make the standardization program operable were identified. The assessors expressed needs for ‘harmonization of practices among laboratories’, ‘revising the standards and related guidelines’, ‘adopting a staged and continuous approach for standardization due to the wide heterogeneity in the laboratories’, ‘recognizing different levels of quality improvement among the laboratories and formulating different operational plans according to laboratories’ resources’.

Interestingly, similar solutions regarding the standardization program and it’s standards were explicitly considered by the laboratories’ directors including ‘introducing strategies to raise better understanding of the standardization process’, ‘adopting a stepwise approach’ and a slower and more gradual way of implementation’, ‘revising and updating the standards, their related checklists and the technical guidelines based on the international standards considering the country context and situation’ and ‘learning from experiences of the previous assessment runs’.

Adopting a stepwise approach to gradually overcome the challenges was also suggested in a study conducted in Africa (Guy-Michel et al., 2010) and such stepwise models have already been implemented successfully in Thailand, Argentina and Kenya (Wattanasri, Manoroma, & Viriyayudhagorn, 2010; Fundación Bioquímica Argentina, 2010; Zeh et al., 2010). Furthermore, some studies suggested that local circumstances and different levels of quality improvement among the laboratories should be taken into account as they have been mainly caused by differences in laboratories’ resources (Ahmad, Ahmad Khan, & Atif Ahmad, 2009; Wattanasri et al., 2010; Safadel et al., 2013; Datema et al., 2011).

### 3.2 The Assessment Process and Assessor

The second set of aspects considered problematic was related to the assessment process and assessors.

The assessors mentioned some challenges regarding the assessment process. ‘Shortage of assessors, assessors’ dissatisfaction’ and ‘the lack of incentives (financial and non-financial) for the assessors’, ‘difficulties in the standards’ interpretation by assessors’ and ‘inadequate assessor training’ were important perceived challenges. The issue of the staff shortage was also mentioned in studies conducted in Pakistan and Kenya (Ahmad et al., 2009; Zeh et al., 2010,). Similarly, McGroder and Crawford highlighted challenges with interpretation of the standards in their study in Jamaica (McGrowder et al., 2010).

Assessors mentioned that the assessment of hospital laboratories had been more complex. The multidisciplinary nature of hospital performance, different shifts of work, the diversity in laboratory tests and emergency laboratory tests were issues that make the compliance with standards more difficult compared with outpatient laboratories.

Differences in assessing public and private laboratories due to differences in management, financing and complying with standards were also considered as challenges by the assessors. While the license of private laboratories is renewed based on the accreditation results, such a process is not necessarily applied to public laboratories. So the lack of motivation plays a key role in making the assessment process problematic in public sector resulting in the low acceptance due to the lack of compulsory licensing. In two studies conducted in Ghana and Jordon, the issue of mandating laboratories to be licensed has been also pointed (Cobbina et al., 2012; Qutishat, 2009).

Factors considered by laboratories directors as major challenges of the assessment process and assessor included ‘lack of a shared understanding of the assessment concepts and purposes among the assessors’, the low number of experienced assessors’, ‘inadequate time devoted for each assessment’, ‘long gap between two consecutive assessments’, ‘lack of providing feedback or delayed feedback to the laboratories’, and ‘assessors’ difficulties in communicating with laboratories’, ‘over strict assessments’, ‘inconsistent assessment results by different assessors’, and ‘unfair assessments’.

The assessors believed that the reason behind the inadequate number of assessors and their dissatisfaction is mainly the lack of a formal incentive system. Also, another challenge for the assessors was the differences in assessing public/private and outpatient/hospital laboratories. Differences in assessing public and private laboratories due to different managerial structure and financing resources were also noted in a study conducted in Iran (Anjarani et al., 2013). Conversely, the principle concerns for the laboratory directors were issues related to the inconsistency of the assessment and time related issues of the assessments. Inadequate provision of timely, fair and constructive feedback was also emphasized. Huisman shows that different assessments may lead to different outcomes which can be frustrating for laboratories (Huisman, 2012).

The assessors mentioned main solutions that help to handle the challenges of the assessment process and assessors. The proposed solutions included ‘clearly defined assessor selection criteria (including personal attributes, knowledge and experience considering assessment expertise and technical experience in laboratory)’, ‘the evaluation of assessment process and assessors based on required professional (including managerial skills) and personal attributes’, training (both initial and ongoing training) to ensure the sound understanding of the standards and assessment techniques by the assessors and more number of assessors to increase both frequency of assessment and the hours spent by the assessors in the laboratories. Also it has been suggested that assessors’ motivation and commitment be increased and evaluated by both accreditation body and laboratory professionals. So the accreditation body should have data regarding the assessors’ requirements including training, competence and their performance in actual assessment. Finally, more interactions between the assessors and accreditation body, other assessors and laboratory professionals and providing financial rewards and professional recognition for assessors were suggested.

Items proposed by laboratories’ directors as possible solutions for the challenges related to the assessment process and assessors included ‘building an assessment culture and clarifying its concept’, ‘following a well-defined plan for assessments’, ‘devoting more time to the assessment process’, ‘conducting assessments in several phases’, ‘providing timely and fair feedback to the laboratories’ and ‘creating a network among the assessors that may facilitate more coordinated and integrated assessment functions’. Likewise, ‘additional recruitments of competent assessors in terms of knowledge, experience and skills’ were proposed. Ultimately, ‘having fixed members of the assessment team for a specific period of time to assure a more consistent approach to assessment’ was also suggested.

The assessors’ solutions mainly focused on the assessor selection criteria and appraisal as well as training issues and establishing a reward system which were consistent with findings of other studies (Huisman, 2012; Huisman et al., 2007; Fundación Bioquímica Argentina, 2010; Zeh et al., 2010; Ravaghi et al., 2014; Wattanasri et al., 2010) while the laboratories’ directors paid more attention to the well defined plan for assessments and providing feedback resulting in better learning and coping of laboratories with standards.

### 3.3 Laboratories

Assessors considered the following items to be major challenges faced by laboratories: ‘the lack of motivation among laboratory professionals’, ‘few laboratories’ involvement in the standardization’, ‘inadequate information about the quality standards among the laboratories’, ‘lack of support from laboratory senior management’ and ‘the weak quality culture’.

‘Financial and human resources problems in the laboratories’ were one of the most frequently mentioned problematic factors by laboratories directors, followed by ‘low familiarity with the concepts of the standardization and quality improvement among laboratories’, ‘inertia and reluctance to change’, ‘more pressure on the private laboratories than public owned laboratories’. They also pointed ‘high workload, the inadequate number of laboratory personnel and ‘the high rate of personnel turn over’.

The same point of view was held by the assessors and laboratories’ directors regarding the challenges related to laboratories such as laboratories’ low involvement and motivation for the standardization, lack of related information, support and commitment from managers. Pongpiul et al. found similar findings in Thailand (Pongpirul, Sriratanaban, Asavaroengchai, Thammatach-Aree, & Laoitthi, 2006).

The assessors explained that the most important factors contributing to overcome challenges related to laboratories were ‘training of laboratory staff in understanding the importance of quality concept and issues’, ‘building a culture of quality in laboratories’ and ‘the harmonization among laboratories’.

‘More commitment and involvement from laboratory directors regarding the quality improvement and standardization’, ‘the development of a motivation system to enhance the engagement of laboratories’ staff’, ‘training the staff or directors particularly in the domains of documentation’, ‘better communication with other laboratories to exchange experience’, ‘establishing an internal audit system in laboratories to identify the nonconformities’ and ‘participating in the external quality control program’ were solutions proposed by the laboratories’ directors to handle the challenges related to laboratories. The issues of the top managers’ commitment and rewarding systems to motivate laboratories were also suggested in studies conducted in the African Region and Thailand, respectively (Guy-Michel et al., 2010; Wattanasri et al., 2010). The establishment of the internal audit system is similar to the findings of a study conducted by Cobbina et al. (Cobbina et al., 2012).

### 3.4 External Entities

Items considered by the assessors as problematic issues related to the entities included ‘limited authorities of the GDLA’, ‘low involvement of professional association in the standardization’ and ‘low executive authority of the GDLA’.

The laboratories’ directors mentioned challenges regarding the external entities including ‘discrimination against private laboratories by the entities responsible for the accreditation’, ‘the lack of an executive authority, unwillingness and unclear role of professional associations in the accreditation’.

Solutions suggested by the assessors regarding the external entities included ‘more involvement of professional associations in accreditation process including composition and revision of standards and guidelines’, ‘setting criteria for assessor selection, evaluation of assessment process, and training’. Also, ‘providing educational courses on quality improvement by universities of medical sciences in order to train competent assessors and laboratories staff’ and ‘adding the principles of quality management system to the academic curriculum of laboratory sciences’ were suggested by the assessors.

Laboratories’ directors suggested that professional associations should play a more critical role in the standardization process. Solutions proposed by laboratories’ directors regarding external entities were as follows: ‘the development of incentive mechanisms by the RHL to encourage the accredited laboratories and support them technically or even financially’, ‘the establishment of a coalition and an effective communication among the professional associations and their convergence to make more unified and integrated roles’, ‘more participation of the professional associations in training and empowering the laboratories’ staff and assessors’, revising standards and checklists, ‘the delegation of the assessment process to professional associations’, ‘providing technical assistance to laboratories’, and helping to build a culture of quality’. Also, the laboratories directors suggested that ‘the professional associations play a more eminent role in setting the national laboratory tariff, the quality control of apparatus, and equipment and consumable kits and their pricing’. Their role in ‘the external quality control and calibration’ was also emphasized.

With regard to the external entities, both groups expressed concern regarding low involvement of the professional associations and agreed on the necessity of higher involvement of such associations in the standardization process, in general and in training, in particular. Meanwhile, the laboratories’ directors had keen interest in the delegation of the assessment process to professional associations. The benefits of the participation of professional associations in selecting assessors and developing standards and guidelines were also discussed in studies conducted in European Union countries (Huisman et al., 2007; Huisman, 2012).

### 3.5 Contextual Factors

The assessors agreed that certain issues related to contextual factors were problematic. ‘Low laboratory tariff levels in Iran’, ‘inadequate policy-level support for accreditation’, ‘financial problems, high inflation rate and increased price of laboratory equipment ’ were the most important examples. ‘Financial and economic issues’, ‘lack of timely reimbursement to laboratories by the insurance organizations’, ‘high price of proper kits and equipment’, ‘costs of maintenance services and the space and facilities needed to comply with the requirements of the standards’ were the contextual issues mentioned by the laboratories’ directors

The following solutions were proposed by the assessors regarding contextual issues: ‘policy-level support for laboratory accreditation system’ and ‘reasonable increase of laboratory tariff levels’.

The laboratories’ directors proposed suggestions regarding contextual related issues such as ‘setting reasonable laboratory tariffs’, ‘providing financial aid for the quality improvement program’ and ‘allocating more budgets to medical laboratories’.

The contextual related challenges of standardization from both groups’ viewpoints appear to be more similar, including low laboratory tariff levels, financial problems and inadequate policy-level support for accreditation. Solutions proposed by both groups also mainly focused on the reasonable increase of laboratory tariff levels and the policy-level support of both standardization process and laboratories. The necessity of policy-level support for laboratory accreditation was also highlighted in studies conducted in Serbia and Thailand (Gligic, 2008; Wattanasri et al., 2010). Also, in a study by Ravaghi et al. the issue of lack of full support has been considered as a barrier affecting staff engagement with the quality improvement initiatives (Ravaghi et al., 2014).

## 5. Conclusion

This study provides insights into the attitudes towards the standardization and standards, the assessment process and assessor, laboratories, external entities and contextual factors across two professional groups. It also highlights two perceived challenges and solutions to implement national standards. These two factors are closely related and should be considered when implementing standards since a positive perception of them increases the likelihood of successful standardization. Similarities and divergences regarding challenges and solutions of the standardization within and across medical laboratory directors and assessors, in turn, can provide insights into how this process can be improved. This study may be a useful step to identify current gaps and suggest required interventions. The suggestions introduced in this study deserve policy makers’ attention at both national and local level to continue the progress.

## References

[1] Ahmad M, Ahmad Khan F, Atif Ahmad S (2009). Standardization of pathology laboratories in Pakistan: Problems and prospects. Clinical Biochemistry.

[2] Anjarani S, Safadel N, Dahim P, Amini R, Mahdavi S, Mirab Samiee S (2013). Establishment of National Laboratory Standards in Public and Private Hospital Laboratories. Iranian Public Health.

[3] Aoyagi T, Kawai T (2006). Validation of the ISO 15189 trial assessment results of clinical laboratories-effects of accreditation and interpretation of ISO 15189. Rinsho Byori.

[4] Cobbina E, Agbezudor J. Y, Amuzu P. S, Gyampomah T. K (2012). The current status and future of medical laboratory quality regulation and accreditation in Ghana. Accred Qual Assur.

[5] Dahim P, Amini R, Safadel N, Rashed Marandi F, Khodaverdian K, Rahnamaye Farzami M (2009). Implementation of quality management system in Iranian medical laboratories. Iranian J Public health.

[6] Datema T. A. M, Oskam L, Klatser P. R (2011). Review and comparison of quality standards, guidelines and regulations for laboratories. Afr J Lab Med.

[7] Gershy-Damet G, Rotz P, Cross D, Belabbes E, Cham F, Ndihokubwayo J, Nkengasong J (2010). The World Health Organization African Region Laboratory Accreditation Process Improving the Quality of Laboratory Systems in the African Region. Am J Clin Pathol.

[8] Gligic L (2008). Status of development and implementation of medical laboratories accreditation in Serbia. Jmb.

[9] Gough L, Reynolds T (2000). Is clinical pathology accreditation worth it? A survey of CPA-accredited laboratories. Clin Perform Qual Health Care.

[10] Greenfield D, Braithwaite B (2008). Health sector accreditation research: a systematic review. INT J QUAL HEALTH C.

[11] Huisman W (2012). European medical laboratory accreditation. Present situation and steps to harmonization. Clin Chem Lab Med.

[12] Huisman W, Horvath A. R, Burnett D, Blaton V, Czikkely R, Jansen R, Zerah S (2007). Accreditation of medical laboratories in the European Union. Clin Chem Lab Med.

[13] McGrowder D, Crawford T, Irving R, Brown P, Anderson-Jackson L (2010). How prepared are medical and non-medical laboratories in Jamaica for accreditation?. Accred Qual Assur.

[14] Nkengasong J. N (2010). A Shifting Paradigm in Strengthening Laboratory Health Systems for Global Health. American Journal of Clinical Pathology.

[15] Plebani M (2001). Role of inspectors in external review mechanisms: criteria for selection, training and appraisal. Clinica Chimica Acta.

[16] Pongpirul K, Sriratanaban J, Asavaroengchai S, Thammatach-Aree J, Laoitthi P (2006). Comparison of health care professionals’ and surveyors’ opinions on problems and obstacles in implementing quality management system in Thailand: a national survey. Int J Qual Health Care.

[17] Programa de Acreditación de Laboratorios, Conclusiones Fundación Bioquímica Argentina Web site.

[18] Qutishat A. S (2008). Medical laboratory quality and accreditation in Jordan. Clin Biochem.

[19] Ravaghi H, Abolhassani N, Dahim P, Shaarbafchi N, Anjarani N, Safadel N (2014). Laboratory accreditation assessors `attitudes towards and experiences of national quality standards: a qualitative study in Iran. Accreditation and Quality Assurance.

[20] Ravaghi H, Khodayari Zarnaq R, Adel A, Badpa M, Adel M, Abolhassani N (2014). A Survey on Clinical Governance Awareness Among Clinical Staff: A Cross-Sectional Study. Global Journal of Health Science.

[21] Safadel N, Dahim P, Anjarani S, Rahnamaye Farzami M, Mirab Samiee S, Amini R, Rashed Marand F (2013). Challenges of Implementing Iranian National Laboratory Standards. Iranian J Public Health.

[22] Spitzenberger F, Edelhäuser R (2006). Accreditation of Medical Laboratories in Europe: Statutory Framework, Current Situation and Perspectives. Transfus Med Hemother.

[23] Wattanasri N, Manoroma W, Viriyayudhagorn S (2010). Laboratory Accreditation in Thailand: A Systemic Approach. Am J Clin Pathol.

[24] Zeh C. E, Inzaule S. C, Magero V. O, Thomas T. K, Laserson K. F, Hart C. E, Nkengasong J. N (2010). Field Experience in Implementing ISO 15189 in Kisumu, Kenya. Am J Clin Pathol.

